# Increased risk of deep vein thrombosis and pulmonary thromboembolism in patients with aortic aneurysms: A nationwide cohort study

**DOI:** 10.1371/journal.pone.0178587

**Published:** 2017-06-07

**Authors:** Feng-You Lee, Wei-Kung Chen, Chun-Hsiang Chiu, Cheng-Li Lin, Chia-Hung Kao, Chao-Hsien Chen, Tse-Yen Yang, Ching-Yuan Lai

**Affiliations:** 1 Department of Emergency Medicine, Taichung Tzu Chi Hospital, Buddhist Tzu Chi Medical Foundation, Taichung, Taiwan; 2 Department of Emergency Medicine, Trauma and Emergency Center, China Medical University Hospital, Taichung, Taiwan; 3 Division of Infectious Diseases and Tropical Medicine, Department of Internal Medicine, Tri-Service General Hospital, National Defense Medical Center, Taipei, Taiwan; 4 Management Office for Health Data, China Medical University Hospital, Taichung, Taiwan; 5 College of Medicine, China Medical University, Taichung, Taiwan; 6 Graduate Institute of Clinical Medical Science and School of Medicine, College of Medicine, China Medical University, Taichung, Taiwan; 7 Department of Nuclear Medicine and PET Center, China Medical University Hospital, Taichung, Taiwan; 8 Department of Bioinformatics and Medical Engineering, Asia University, Taichung, Taiwan; 9 Department of Medical Laboratory Science and Biotechnology, China Medical University, Taichung, Taiwan; 10 Department of Medical Research, China Medical University Hospital, China Medical University, Taichung, Taiwan; Medical University Innsbruck, AUSTRIA

## Abstract

Studies on the association between aortic aneurysm (AA) and the subsequent risk of venous thromboembolism (VTE) are limited to a few case reports and investigations which only focused on surgical effects. Therefore, we used the National Health Insurance Research Database to clarify whether patients with AAs have a heightened risk of subsequent VTEs, including deep vein thrombosis (DVT) and pulmonary embolism (PE). Our retrospective cohort study comprised patients aged ≥ 18 years who received a diagnosis of an AA and were hospitalized at any point during 2000–2010 (n = 16,630). Each AA patient was frequency-matched to 4 non-AA hospitalized patients based on age, sex, and index year (n = 66,453). The Cox proportional hazard regressions model was used to estimate the adjusted effect of AAs on VTE risk. The overall incidence of DVT and PE was higher in the patients with AA than in the non-AA group patients (23.5 versus 13.2 and 13.5 versus 7.98/1,000 person-years). After adjustment for age, sex, duration of hospitalization in the study period, and comorbidities, patients with AAs were associated with a 1.88-fold higher risk of DVT and 1.90-fold higher risk of PE compared to the non-AA cohort. Patients with abdominal AAs were more likely to develop DVT, whereas thoracic AA patients were more likely to develop PE. A diagnosis of a ruptured AA was associated with a substantially increased risk of DVT. Surgical treatment of AAs was associated with a heightened risk of VTE within 6-months post-operation. Our study demonstrates that AAs are associated with an increased risk of subsequent VTE. Future investigations are encouraged to delineate the mechanisms underlying this association and to evaluate the cost-effectiveness of screening for VTEs in patients with AAs.

## Introduction

An aortic aneurysm (AA) is defined as a loss of aortic wall integrity, which results in the permanent and irreversible focal dilatation of vessel walls [1, 2]. Most AAs are silent until they dissect or rupture, leading to life-threatening complications [3, 4]. In the United States, approximately 4,500 patients die of ruptured abdominal AAs (AAAs) annually and another 1,400 die from reparative and preventive repair surgery, creating a burden to the health care system [1, 5].

Venous thromboembolisms (VTEs), comprising deep vein thrombosis (DVT) and pulmonary embolisms (PEs), are a relatively common cause of death worldwide. The overall age- and gender-adjusted annual incidence is 117 per 100,000 population [6, 7]. DVT involves the formation of blood clots in the deep venous system and frequently causes swelling, pain, a lukewarm sensation, and venous engorgement of the lower extremities. Approximately 40%-50% of cases progress to PE, with a mortality rate of 30% in the absence of early recognition and prompt intervention [6, 8]. Widely accepted predisposing factors for VTEs can be grouped into 3 categories, termed the “Virchow’s triad”: endothelial injury, stasis of blood flow and, hypercoagulability [9].

Cardiovascular disease remains the major cause of death among AA patients, regardless of whether intervention therapy for aneurysms is administered [10]. VTE is ranked as the third most common form of cardiovascular disease, after ischemic heart disease and cerebral vascular disease [6, 8, 11]. Because the characteristics of aneurysms include immune response, inflammation, an activated coagulation system, and possible mass-compression effects, all of which promote thrombus formation, we hypothesized that patients with AAs would bear a higher risk of VTE than that of patients without AAs. Therefore, we conducted an extensive database analysis to clarify the possible association between AAs and the risk of VTEs.

## Methods

The designated statements of text were based on the reporting items of the REporting of studies Conducted using Observational Routinely collected health Data (RECORD) checklist (http://www.record-statement.org). The RECORD checklist of observational studies which extended from STROBE statement (http://strobe-statement.org) are available as supporting information in S1 RECORD Checklist.

### Data source

This study used data from the National Health Insurance Research Database (NHIRD). The National Health Research Institutes (NHRI) organizes the NHIRD, which contains all claims data from the Taiwan National Health Insurance (NHI) program. The Taiwanese government established the NHI in 1995 as a single-payer, compulsory program for all 23 million Taiwanese citizens. The data from the NHIRD includes a registry of beneficiaries and all medical services administered. In this study, histories of disease diagnosis were obtained from inpatient files. These files record diseases according to the International Classification of Diseases, Ninth Revision, Clinical Modification (ICD-9-CM). To protect patient privacy, the NHRI removes identifying information and assigns an anonymous number before releasing patient records for research. This study was approved by the Ethics Review Board of China Medical University (CMUH104-REC2-115).

### Study population

This study focused on the risk of DVT and PE in individuals with and without AAs. Based on the inpatient claims, we sorted patients into a cohort of patients with AAs and a non-AA cohort without AAs. The AA cohort included adult patients with newly diagnosed AA during hospitalization (ICD-9-CM 441.1–441.9) between January 1, 2000 and December 31, 2010, with the index date set as the date of first diagnosis. For each patient with AAs, four hospitalized patients without AAs from NHIRD were randomly selected for the non-AA cohort. The index date for non-AA patients was randomly appointed a month and a day with the same index year of the matched AA cases. The non-AA cohort was frequency matched with the AA cohort by same distributions over strata of sex, age (every 5-y span), and index year. We excluded individuals with histories of DVT (ICD-9-CM 453.8) and PE (ICD-9-CM 415.1, excluded 415.11) before the index date. This study had 2 major outcomes: DVT and PE events. The patients were divided into 2 paired observation groups. In the first group, patients were followed until they unenrolled from the NHI program or experienced DVT; in the second group, patients were observed until they unenrolled from the NHI program or developed PE. Observation of the remaining patients from both groups was ended after reaching records from December 31, 2011.

A history of cancer [ICD-9-CM 140–208], heart failure [ICD-9-CM 428], atrial fibrillation [ICD-9-CM 427.31], hypertension [ICD-9-CM 401–405], cerebral vascular accident [CVA, ICD-9-CM 430–438], diabetes [ICD-9-CM 250], pregnancy [ICD-9-CM 640.x1–676.x1, 640.x2–676.x2 and 650–659 and ICD-9-procedure 72–74] and lower leg fracture or surgery [ICD-9-CM 820, 821, 823, ICD-9-procedure 81.51–81.54]) before the endpoints were included as a time-dependent covariate for comorbidities.

We also considered the effects of different types of AA. AA patients were classified into thoracic AAs (TAAs, ICD-9-CM 441.1 and 441.2), AAAs (ICD-9-CM 441.3 and 441.4) and others (ICD-9-CM 441.5–441.9). Another classification of AA was based on the presence of rupture (ICD-9-CM 441.1, 441.3, 441.5, and 441.6) or its absence (ICD-9-CM 441.2, 441.4 and 441.7–441.9). To take into account the effect of surgical procedures, intervention with surgery (ICD-9-CM procedure codes 38.34, 38.44, 38.45, 38.64, 39.52) in AA patients was calculated in our study.

### Statistical analysis

To compare the 2 cohorts, we present the mean and standard deviation (SD) for patient ages and duration of hospitalization, and the numbers and proportions of sex and comorbidities; we additionally applied a *t* test to compare ages and duration of hospitalization, and a chi-squared test for sex and comorbidities. We calculated the events number, person-years, and incidence density of DVT and PE in each group, and estimated the cumulative incidence curves for the 2 study cohorts through the Kaplan–Meier method. We also tested the curve difference between the 2 study cohorts through a log-rank test. We evaluated the risk of DVT and PE for AA patients compared with non-AA patients by using multivariable Cox proportional hazard models with results presented as adjusted hazard ratios (HRs) and 95% confidence intervals (CIs). We analyzed the risk of DVT and PE among AA and non-AA patients stratified by age, sex, and comorbidities. We also assessed the risk of DVT and PE among different types of AA by using multivariable Cox proportional hazard models. To address the concern of constant proportionality, we examined the proportional hazard model assumption using a test of scaled Schoenfeld residuals. The results showed that there was no significant relationship between Schoenfeld residuals for aortic aneurysm and follow-up time (p-value = 0.17) in the model evaluating the DVT risk. In the model evaluating the PE risk throughout overall follow-up period, results of the test revealed a significant relationship between Schoenfeld residuals for aortic aneurysm and follow-up time (p-value = 0.01), suggesting the proportionality assumption was violated. In the subsequent analyses, we stratified the follow-up duration to deal with the violation of proportional hazard assumption. We also applied the threshold regression as the sensitivity analysis to estimate the risk of VTE, DVT and PE between AA and non-AA cohort [12]. There were 2 models to describe the risk of VTE, DVT and PE: 1) regression for the logarithm for the baseline disease occurrence; 2) regression for the disease process rate. All statistical analyses were performed using SAS 9.4 software (SAS Institute, Cary, NC, USA) and the incidence curve was determined with R software (R Foundation for Statistical Computing, Vienna, Austria). We set the significance level at ≤ 0.05 for a 2-sided test.

## Results

We assembled a cohort of 16,630 AA patients and a non-AA cohort with a 4–fold number of hospitalized patients (Table 1). The difference in the distributions of age and sex between the AA cohort and non-AA cohort were nonsignificant because of the frequency matching at baseline. Most participants in both cohorts were older than 75 years (56.4%) and male (73.2%). The duration of hospitalization was longer for AA patients than non-AA subjects (57.9 vs. 37.0 days) in the study period. The prevalence of comorbidity, including heart failure and all cancer, were significantly higher in the AA cohort than in the non-AA cohort.

**Table 1:** Comparison of demographics and comorbidity between aortic aneurysm patients and controls.

	Aortic aneurysm		*p*-value
	Yes (N = 16630) n(%)	No (N = 66453) n(%)	
Age, years			0.99
≤64	2953(17.8)	11797(17.8)	
65–74	4300(25.9)	17192(25.9)	
≥75	9377(56.4)	37464(56.4)	
Mean (SD) ^†^	73.6(12.4)	74.1(12.4)	0.001
Gender			0.99
Female	4449(26.8)	17782(26.8)	
Male	12181(73.3)	48671(73.2)	
Duration of hospitalization in the study period, Mean (SD) ^†^	57.9(121.2)	37.0(109.6)	<0.001
Comorbidity			
Hypertension	12402(74.6)	49308(74.2)	0.32
Diabetes	3964(23.8)	17674(26.6)	<0.001
CVA	6184(37.2)	25408(38.2)	0.01
Heart failure	4631(27.9)	17419(26.2)	0.001
All cancer	2567(15.4)	9493(14.3)	<0.001
Pregnancy	48(0.29)	211(0.32)	0.55
Atrial fibrillation	2237(13.5)	9053(13.6)	0.56
Lower leg fracture or surgery	2171(13.1)	9867(14.9)	<0.001

CVA denotes cerebral vascular disease; Chi-square test examined categorical data;

^†^T-test examined continuous;

**Fig 1:**
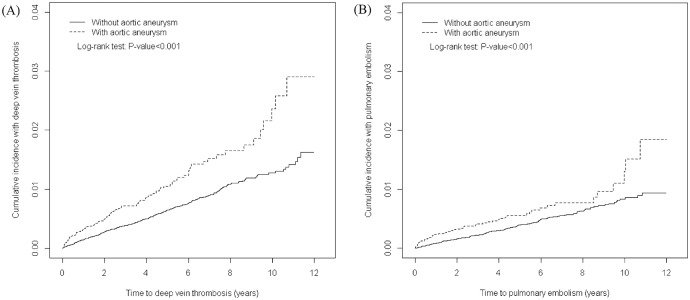
Cumulative incidence of (A) deep vein thrombosis (DVT) and (B) pulmonary embolism (PE) in patients with aortic aneurysms and patients without aortic aneurysms.

We observed that the AA cohort developed 113 DVT events and 65 PE events and the non-AA cohort developed 387 DVT events and 235 PE events (Table 2). Fig 1A and 1B both show that the cumulative incidences of DVT and PE were significantly higher for the AA cohort than for the non-AA cohort (log-rank test p < 0.001). Regarding the overall risk of VTE between AA and non-AA subjects, the AA patient had a 1.89-fold higher risk of VTE than the non-AA subjects (HR = 1.89, 95% CI = 1.58–2.25). Adjusted for age, sex, duration of hospitalization in the study period, and comorbidities, the AA patients had a 1.88-fold and 1.90-fold increased risk of DVT (HR = 1.88, 95% CI = 1.52–2.33) and PE (HR = 1.90, 95% CI = 1.43–2.51) compared with the non-AA cohort, respectively.

**Table 2:** Incidence and adjusted hazard ratio of DVT and PE by sex, age and comorbidity for aortic aneurysm patients compared to controls.

Variables	Aortic aneurysm						Compared to Control	
	Yes			No				
	Events n	PY	Rate^#^	Events n	PY	Rate^#^	Crude HR (95% CI)	Adjusted HR (95% CI)
VTE^a^	168	47991	35.0	598	293784	20.4	1.70(1.43, 2.01)***	1.89(1.58, 2.25)***
DVT								
All^a^	113	48081	23.5	387	294111	13.2	1.76(1.43, 2.18)***	1.88(1.52, 2.33)***
Gender^b^								
Female	38	13642	27.9	111	81157	13.7	1.99(1.37, 2.87)***	2.19(1.51, 3.20)***
Male	75	34438	21.8	276	212954	13.0	1.67(1.29, 2.15)***	1.75(1.35, 2.28)***
Age, years^c^								
≤64	27	12571	21.5	52	64393	8.08	2.64(1.66, 4.21)***	2.45(1.54, 3.94)***
65–74	34	14813	23.0	110	88312	12.5	1.83(1.24, 2.69)**	1.86(1.26, 2.75)**
≥75	52	20696	25.1	225	141407	15.9	1.57(1.16, 2.12)**	1.70(1.25, 2.32)***
Comorbidity^§^^d^								
No	14	4559	30.7	13	40863	3.18	9.12(4.27, 19.5)***	12.0(5.03, 28.7)***
Yes	99	43522	22.8	374	253248	14.8	1.53(1.22, 1.91)***	1.63(1.30, 2.04)***
PE								
All^a^	65	48234	13.5	235	294626	7.98	1.67(1.27, 2.20)***	1.90(1.43, 2.51)***
Gender^b^								
Female	19	13685	13.9	84	81218	10.3	1.31(0.80, 2.16)	1.64(0.99, 2.73)
Male	46	34549	13.3	151	213409	7.08	1.87(1.34, 2.60)***	2.06(1.47, 2.89)***
Age, years^c^								
≤64	10	12628	7.92	18	64509	2.79	2.87(1.33, 6.23)**	3.32(1.48, 7.47)**
65–74	20	14852	13.5	65	88516	7.34	1.84(1.12, 3.05)*	1.81(1.09, 3.01)*
≥75	35	20754	16.9	152	141601	10.7	1.55(1.07, 2.24)*	1.78(1.22, 2.59)**
Comorbidity^§^^d^								
No	1	4604	2.17	9	40897	2.20	0.93(0.12, 7.43)	1.11(0.11, 11.1)
Yes	64	43630	14.7	226	253729	8.91	1.63(1.23, 2.15)***	1.83(1.38, 2.43)***
Follow-up time^a^								
<2	40	22278	18.0	94	118132	7.96	2.21(1.52, 3.19)***	3.20(2.18, 4.71)***
≥2	25	25956	9.63	141	176494	7.99	1.22(0.80, 1.87)	1.30(0.85, 2.00)

PY, person-years; Rate^#^, incidence rate, per 10,000 person-years; Crude HR: relative hazard ratio;

^a:^ adjusted hazard ratio controlling for age, sex, duration of hospitalization in the study period, and comorbidities of hypertension, diabetes, CVA, heart failure, all cancer, pregnancy, atrial fibrillation, and lower leg fracture or surgery;

^b:^ adjusted hazard ratio controlling for age, duration of hospitalization in the study period, and comorbidities of hypertension, diabetes, CVA, heart failure, all cancer, pregnancy, atrial fibrillation, and lower leg fracture or surgery;

^c:^ adjusted hazard ratio controlling for sex, duration of hospitalization in the study period, and comorbidities of hypertension, diabetes, CVA, heart failure, all cancer, pregnancy, atrial fibrillation, and lower leg fracture or surgery;

^d:^ adjusted hazard ratio controlling for age and sex;

Comorbidity^§^: Patients with any one of the comorbidities hypertension, diabetes, CVA, heart failure, all cancer, pregnancy, atrial fibrillation, and lower leg fracture or surgery were classified as the comorbidity group

*p<0.05,

**p<0.01,

***p<0.001

Table 2 also shows the risk of DVT and PE stratified by gender, age, and comorbidities. The HRs for DVT were 2.19 (95% CI = 1.51–3.20) and 1.75 (95% CI = 1.35–2.28) for the female and male AA patients, respectively. When the AA patients were compared with the non-AA patients, the risk of DVT was highest in those aged ≤64 years (HR = 2.45, 95% CI = 1.54–3.94) followed by patients aged 65–74 years (HR = 1.86, 95% CI = 1.26–2.75) and ≥ 75 years (HR = 1. 70, 95% CI = 1.25–2.32). The risk of DVT was significantly higher for individuals with AAs than those without AAs, and was stratified by individuals without any comorbidity (HR = 12.0, 95% CI = 5.03–28.7) and by individuals with at least one comorbidity (HR = 1.63, 95% CI = 1.30–2.04). The risk of PE for the AA patients compared with the non-AA ones was significantly higher in each age group. Male AA patients had a 2.06-fold higher risk of PE than male patients from the non-AA cohort did (HR = 2.06, 95% CI = 1.47–2.89), but differences were not statistically significant among female patients (HR = 1.64, 95% CI = 0.99–2.73). The HR of PE was 1.83 (95% CI = 1.38–2.43) among the AA patients with at least one comorbidity than among the non-AA patients. In the first two years of follow-up, the AA cohort had a higher risk of PE compared with the non-AA cohort (HR = 3.20, 95% CI = 2.18–4.71).

After adjustment for age, sex, duration of hospitalization in the study period, and comorbidities (Table 3), the HRs of DVT were 1.85 (95% CI = 1.28–2.66) and 2.07 (95% CI = 1.57–2.72) for TAA and AAA patients, respectively. The DVT risks were 3.81 (95% CI = 2.30–6.31) and 1.74 (95% CI = 1.38–2.18) for the AA patients with and without rupture, respectively.

**Table 3:** Comparisons of incidence, and hazard ratios of DVT and PE by subtypes of aortic aneurysm.

Variables	N	Event	Rate^#^	Crude HR (95% CI)	Adjusted HR^†^ (95% CI)
DVT					
Non-Aortic aneurysm	66453	387	13.2	1(Reference)	1(Reference)
Thoracic aneurysm	4691	32	22.5	1.69(1.18, 2.42)***	1.85(1.28, 2.66)***
Abdominal aneurysm	8596	62	26.5	1.98(1.52, 2.60)***	2.07(1.57, 2.72)***
Others	3343	19	18.2	1.37(0.86, 2.17)	1.48(0.93, 2.35)
Non-Aortic aneurysm	66453	387	13.2	1(Reference)	1(Reference)
With Rupture	2141	16	38.3	2.85(1.73, 4.69)***	3.81(2.30, 6.31)***
Without Rupture	14489	97	22.1	1.66(1.33, 2.07)***	1.74(1.38, 2.18)***
PE					
Non-Aortic aneurysm	66453	235	7.98	1(Reference)	1(Reference)
Thoracic aneurysm	4691	20	14.0	1.74(1.10, 2.74)*	2.03(1.28, 3.22)**
Abdominal aneurysm	8596	33	14.0	1.73(1.20,2 .50)**	1.96(1.35, 2.84)**
Others	3343	12	11.5	1.42(0.80, 2.54)	1.60(0.89, 2.86)
Non-Aortic aneurysm	66453	235	7.98	1(Reference)	1(Reference)
With Rupture	2141	5	11.9	1.46(0.60, 3.53)	2.37(0.97, 5.77)
Without Rupture	14489	60	13.6	1.69(1.27, 2.24)***	1.86(1.40, 2.49)***

Rate^#^, incidence rate, per 10,000 person-years; Crude HR: relative hazard ratio;

Adjusted HR^†^: adjusted hazard ratio controlling for age, sex, duration of hospitalization in the study period, and comorbidities of hypertension, diabetes, CVA, heart failure, all cancer, pregnancy, atrial fibrillation, and lower leg fracture or surgery;

ICD-9-CM: Thoracic aneurysm: 441.1 and 441.2; Abdominal aneurysm: 441.3 and 441.4; Others: 441.5, 441.6, 441.7, 441.8 and 441.9; Rupture: 441.1, 441.3, 441.5, 441.6; Without Rupture: 441.2, 441.4, 441.7, 441.8, 441.9

*p<0.05,

**p<0.01,

***p<0.001

The estimated PE risk revealed a 2.03-fold (95% CI = 1.28–3.22) and 1.96-fold (95% CI = 1.35–2.84) increased risk of PE for the TAA and AAA patients, respectively. The results showed a significantly higher PE risk for AA patients without rupture (HR = 1.86, 95% CI = 1.40–2.49) than for non-AA patients. Patients who underwent surgery for AAs had a higher risk for VTE (HR = 2.21, 95% CI = 1.64–2.99) than it was in the non-AA cohort (Table 4).

**Table 4:** Comparisons of incidence, and hazard ratios of VTE by surgery of Aortic aneurysm.

Variables	N	Event	Rate^#^	Crude HR (95% CI)	Adjusted HR^†^ (95% CI)
VTE					
Non-Aortic aneurysm	66453	598	20.4	1(Reference)	1(Reference)
Aortic aneurysm without surgery	12977	120	35.0	1.69(1.39, 2.05)***	1.76(1.44, 2.15)***
Aortic aneurysm with surgery	3653	48	35.0	1.72(1.28, 2.30)***	2.21(1.64, 2.99)***
Post-operation≧3 months	2827	34	25.5	1.26(0.89, 1.78)	1.61(1.13, 2.29)**
Post-operation≧6 months	2679	29	22.0	1.08(0.75, 1.57)	1.38(0.95, 2.02)
Post-operation≧1 year	2520	24	18.4	0.91(0.61, 1.37)	1.16(0.77, 1.76)

Rate^#^, incidence rate, per 10,000 person-years; Crude HR: relative hazard ratio;

Adjusted HR^†^: adjusted hazard ratio controlling for age, sex, duration of hospitalization in the study period, and comorbidities of hypertension, diabetes, CVA, heart failure, all cancer, pregnancy, atrial fibrillation, and lower leg fracture or surgery;

**p<0.01,

***p<0.001

Table 5 showed results of the threshold regression for the risk of VTE, DVT and PE. The results showed AA patents with a negative regression coefficient (-0.33) in ln(y_0_) compared with non-AA individuals, which meant AA patients bore higher risk for VTE at baseline. However, the regression coefficient was positive (0.26) for AA patient in μ and that meant AA patient tends away from the zero threshold of disease occurrence. The results for DVT and PE risk between AA and non-AA cohort were similar with the result for VTE risk.

**Table 5:** Threshold regression output for VTE after aortic aneurysm I: Regression for the logarithm of the baseline impairment ln(y0) = a0+a1X1+…akXk.

	VTE		DVT		PE	
Regression for ln(y_0_)	Coefficient a(SE)	p-value	Coefficient a(SE)	p-value	Coefficient a(SE)	p-value
Aortic aneurysm	-0.33(0.02)	<0.001	-0.10(0.02)	<0.001	-0.52(0.03)	<0.001
Age, years	-0.01(0.00)	<0.001	-0.01(0.00)	<0.001	-0.00(0.00)	<0.001
Gender	-0.23(0.02)	<0.001	-0.21(0.02)	<0.001	-0.18(0.03)	<0.001
Comorbidity						
Hypertension	0.19(0.02)	<0.001	0.02(0.03)	<0.001	0.29(0.03)	<0.001
Diabetes	-0.11(0.02)	<0.001	0.04(0.02)	<0.001	-0.16(0.03)	<0.001
CVA	0.05(0.02)	<0.001	-0.06(0.02)	<0.001	0.23(0.03)	<0.001
Heart failure	-0.25(0.02)	<0.001	-0.30(0.02)	<0.001	-0.08(0.03)	<0.001
All cancer	-0.22(0.02)	<0.001	-0.12(0.03)	<0.001	-0.18(0.03)	<0.001
Pregnancy	0.10(0.28)	<0.001	0.10(0.27)	<0.001	0.14(1.20)	<0.001
Atrial fibrillation	0.30(0.03)	<0.001	0.34(0.03)	<0.001	0.15(0.04)	<0.001
Lower leg fracture or surgery	0.12(0.02)	<0.001	0.10(0.03)	<0.001	-0.02(0.03)	<0.001
Constant a_0_	1.10(0.07)	<0.001	1.18(0.08)	<0.001	1.29(0.11)	<0.001
II: Regression for the impairment process rate μ = b_0_+b_1_X_1_+…+b_k_X_k_						
Regression for rate, μ	Coefficient b(SE)	p-value	Coefficient b(SE)	p-value	Coefficient b(SE)	p-value
Aortic aneurysm	0.26(0.03)	<0.001	0.02(0.04)	<0.001	0.56(0.05)	<0.001
Age, years	0.005(0.001)	<0.001	0.004(0.001)	<0.001	0.01(0.001)	<0.001
Gender	0.24(0.03)	<0.001	0.22(0.03)	<0.001	0.24(0.04)	<0.001
Comorbidity						
Hypertension	-0.29(0.03)	<0.001	-0.11(0.04)	<0.001	-0.44(0.05)	<0.001
Diabetes	0.05(0.03)	<0.001	-0.11(0.03)	<0.001	0.16(0.04)	<0.001
CVA	-0.06(0.02)	<0.001	0.06(0.03)	<0.001	-0.25(0.04)	<0.001
Heart failure	0.11(0.03)	<0.001	0.26(0.03)	<0.001	-0.11(0.04)	<0.001
All cancer	0.17(0.03)	<0.001	0.07(0.04)	<0.001	0.21(0.05)	<0.001
Pregnancy	0.11(0.37)	<0.001	0.11(0.40)	<0.001	0.15(1.71)	<0.001
Atrial fibrillation	-0.31(0.03)	<0.001	-0.39(0.04)	<0.001	-0.15(0.05)	<0.001
Lower leg fracture or surgery	-0.18(0.03)	<0.001	-0.15(0.04)	<0.001	-0.04(0.04)	<0.001
Constant b_0_	0.93(0.09)	<0.001	0.94(0.11)	<0.001	1.07(0.16)	<0.001

SE: standard error

## Discussion

Based on a review of the literature, this is the first nationwide population-based cohort study to investigate whether patients with AAs have a heightened risk of developing VTE, including DVT and PE. The primary results indicate that patients with AAs had a significantly higher overall incidence and crude risk of developing DVT and PE, and that outcomes remained statistically significant after adjustment for the confounders of age, sex, duration of hospitalization, and comorbidity. In contrast to the general population, patients with AAs had a 1.88- and 1.90-fold higher risk of developing DVT and PE, respectively.

Epidemiological investigations conducted between 1951 and 1995 have demonstrated that the incidence and mortality rate of AAs rose steadily in Western developed countries, but have since steeply declined during the twenty-first century, possibly as a result of several public health policies that have reduced the prevalence of hypertension, hyperlipidemia and smoking [13]. Notably, the risk of AA increases dramatically after 60 years of age [1]. Patients older than or equal to 75 years predominated in our AA cohort (56.4%), and males accounted for most cases (73.3%), which is consistent with prior studies [1, 14].

Several epidemiological studies that have examined the incidence rates of VTE among people of European ancestry reported 7.1 to 38.7 per 10,000 person-years [7, 11, 15–28]. In addition, White et al. [28] reported racial and ethnic differences in a California study that the incidence of DVT was highest among African Americans, followed by Caucasians, Hispanics, and Asians. In our study, the calculated incidence of VTE in non-AA cohort is 20.4 per 10,000 person-years, and 35.0 per 10,000 person-years in AA cohort, which is comparable with data from Tecumseh Community Health Study [17], but higher than that from other investigations conducted in the Western world [15, 20, 22, 24, 27, 28]. We suppose that it may be attributed to the highly developed and accessible healthcare systems in Taiwan that the disease might be early detected through frequent outpatient visits and/or widely used radiologic technics. Notably, the true incidence of VTE is difficult to estimate, since a substantial proportion of patients were diagnosed postmortem at autopsy, leading to overall underestimation [22, 29].

The major findings of our study indicate that the patients with AA, in contrast to the non-AA patients, bore a significantly higher risk of DVT, an association that remained evident in each age group, in men and women, and in those with or without comorbidities. The actual mechanism underlying this association is unclear but there are several possible explanations.

First, the pathological features of AAs include degeneration of the tunica media due to degradation and loss of elastic fibers, smooth muscle fibers, and the extracellular matrix; deposition of proteoglycans, the preceding inflammatory reaction, oxidative stress or the immunization processes of connective tissues can accelerate vessel wall degeneration [2, 30, 31]. These properties might induce aggregation of inflammatory cells, secretion of cytokines and activation of the immune system in other venous systems, leading to endothelial damage, platelet aggregation and the production of reactive oxygen species that all contribute to thrombus formation.

Second, an increasing number of patients with AAs underwent elective or emergency repair surgery [1, 32–35]. Surgery-related endothelial damage, inflammation, activation of coagulation, temporary inhibition of fibrinolysis, and immobilization related secondary venous stasis might all increase the risk of DVT. Olin et al. [36, 37] prospectively investigated 50 consecutive AAA surgery patients 5 days after the procedures were performed and found the incidence of DVT to be 18%. Scarborough et al. [38, 39] discovered the incidence of VTEs after open aortic surgery to be 2.4%. Bradbury et al. [40] reviewed 65 patients who survived surgery for a ruptured AAA and reported that 40% of patients developed postoperative venous thrombosis, and Eagleton et al. [41] found the DVT incidence to be 6% among 50 AAA patients after endovascular surgery. By contrast, de Maistre et al. [42] proposed that the delayed use of anticoagulants, blood transfusion and activation of coagulation by the aneurysm itself might contribute to the high frequency of VTEs after AAA surgery. In our subgroup analysis, patients with surgical treatment for AAs were associated with substantially heightened risk for VTE, especially within the first 6 months post-operatively.

Third, an aneurysm mass could compress adjacent venous systems such as the superior and inferior vena cava (IVC) or iliac veins, leading to venous stasis and a heightened risk of thrombus formation. One of the most well-known examples is May–Thurner syndrome, referring to compression of the left iliac vein by the right iliac artery, resulting in venous stasis and edema of the left leg [7]. Mertens et al. [43] reported 2 elderly male patients who presented with unilateral ilio–femoral DVT caused by venous compression by aortic and iliac aneurysms with secondary thrombosis, and suggested that AAA or tumor compression should be ruled out in elderly patients presenting with unexplained DVT [43]. Moore et al. [44] reported one 55-year-old male with an infra-renal AAA that caused 50% compression of the IVC, resulting in unilateral leg DVT. The authors concluded that the mass effect of AAAs lacking significant hemodynamic compromise might still correlate with the formation of DVT. Although large-scale database analyses are scarce, we believe that the mass compression effect plays an essential role in the development of DVT. Furthermore, our data indicate that female AA patients, in contrast to males, have a higher incidence and risk of DVT; this might be explained by the use of hormone replacement therapy or oral contraceptives by some women, which is known to increase the risk of DVT, and is consistent with the previous observation that female AA patients have a higher risk of rupture and other complications than males do [45]. Moreover, AA patients without comorbidities had a substantially higher risk for DVT than those with comorbidities did, demonstrating that without the interference of time-dependent comorbidity factors, AAs have a critical influence on the development of DVT.

PE has been deemed a pathophysiologically progressive process of DVT, and both conditions share the same precipitating factors. Unsurprisingly, the results of our study reveal that patients with AAs had an overall higher risk for PE regardless of age. However, we cannot offer a precise explanation of the possible mechanisms underlying this correlation, or the presence of gender-related differences. Sajjad et al. [46] recently reported a case of a 79-year-old woman who presented with a pulmonary embolism; a computed tomography angiogram revealed an 8.7-cm AAA that compressed and displaced the adjacent IVC, causing a thromboembolism. The authors emphasized the importance of a thorough examination from the trunk to lower extremities once a PE is confirmed, but they admitted that the actual correlation between PEs and AAs remained unclear.

For further analysis, we divided the AA cohort into patients with TAAs, AAAs and others to clarify whether the AA location would influence the outcomes. The results demonstrate that AAAs increase the risk of DVT, whereas patients with TAAs are more likely to develop PE. The reasons are unclear but the anatomical proximity, which has been shown to enhance the aneurysmal mass effect [47], might be responsible for this phenomenon. We conducted a severity analysis by dividing the AA cohort into ruptured and non-ruptured AA subgroups, with ruptured AAs classified as those that were large or rapidly growing. The results show that patients with ruptured AAs are at a significantly higher risk of DVT but not PE. Brandao et al. [48] proposed that large AAs that compress the IVC are closely related to DVT formation, and Pawlaczyk et al. [49] concluded that reparative surgery for ruptured AAs might increase the risk of DVT.

Despite the strengths of our study, such as the use of samples derived from a nationwide large-scale healthcare database covering over 99% of the national population, providing a representative outcome, there are several potential limitations that deserve mention. First, we cannot confirm the causal relationship between AAs and VTEs on the basis of these retrospective observations. Second, all disease definitions were based on ICD-9-CM codes and mis-, under-, or over-coding could not be fully avoided. Although the sensitivity of Taiwan NHIRD has been determined to be 76% to 90% for diagnosis of other illness, like acute myocardial infarction or stroke[50, 51], the sensitivity of diagnosis of DVT and PE was less in Taiwan NHIRD [52], but previous study from Canadian claim database has pointed out that the diagnosis of DVT and PE in adult was more sensitive by using ICD-9-CM [53]. Moreover, since all diagnosis codes were repeatedly scrutinized by physicians, medical reimbursement specialists and peer review, we believe the diagnoses of VTE, AA or others based on ICD-9 codes in this study were highly reliable. Third, the NHIRD lacks numerous types of crucial information, including family history; levels of physical activity; socioeconomic status; body mass index; severity of comorbidities; laboratory data; anticoagulants or hormone replacement therapy records; and essential data of aneurysms, such as size and morphology, all of which might correlate with DVT development. Fourth, our patients were randomly selected from an inpatient claims database, implying that patients with or without AAs who did not require hospitalization during the study period might have been missed, leading to an underestimation of VTE incidence. Finally, because our investigation sampling was based on people living in Taiwan, the outcomes and affiliated inferences might not be applicable to other regions or countries.

## Conclusion

This population-based retrospective cohort study examined the possible association between AA and VTE risk. Future investigations are encouraged to delineate the exact mechanisms underlying this association and evaluate the cost-effectiveness of radiological screening for VTEs in patients with AAs.

## Supporting information

S1 RECORD Checklist RECORD checklist for the reporting of “Increased Risk of Deep Vein Thrombosis and Pulmonary Thromboembolism in Patients with Aortic Aneurysms: A Nationwide Cohort Study”. (DOCX)

## Abbreviations

AA: aortic aneurysm
aHR: adjusted hazard ratio
CI: confidence interval
DVT: deep vein thrombosis
ICD-9-CM: International Classification of Diseases, Ninth Revision, Clinical Modification
NHI: National Health Insurance program
NHIRD: National Health Insurance Research Database
SD: standard deviation
VTE: venous thromboembolism
